# Accessibility of covariance information creates vulnerability in Federated Learning frameworks

**DOI:** 10.1093/bioinformatics/btad531

**Published:** 2023-08-30

**Authors:** Manuel Huth, Jonas Arruda, Roy Gusinow, Lorenzo Contento, Evelina Tacconelli, Jan Hasenauer

**Affiliations:** Institute of Computational Biology, Helmholtz Munich, Neuherberg 85764, Germany; Life and Medical Sciences Institute, Faculty of Mathematics and Natural Sciences, University of Bonn, Bonn 53115, Germany; Life and Medical Sciences Institute, Faculty of Mathematics and Natural Sciences, University of Bonn, Bonn 53115, Germany; Institute of Computational Biology, Helmholtz Munich, Neuherberg 85764, Germany; Life and Medical Sciences Institute, Faculty of Mathematics and Natural Sciences, University of Bonn, Bonn 53115, Germany; Life and Medical Sciences Institute, Faculty of Mathematics and Natural Sciences, University of Bonn, Bonn 53115, Germany; Division of Infectious Diseases, Department of Diagnostics and Public Health, University of Verona, Verona 37124, Italy; Life and Medical Sciences Institute, Faculty of Mathematics and Natural Sciences, University of Bonn, Bonn 53115, Germany

## Abstract

**Motivation:**

Federated Learning (FL) is gaining traction in various fields as it enables integrative data analysis without sharing sensitive data, such as in healthcare. However, the risk of data leakage caused by malicious attacks must be considered. In this study, we introduce a novel attack algorithm that relies on being able to compute sample means, sample covariances, and construct known linearly independent vectors on the data owner side.

**Results:**

We show that these basic functionalities, which are available in several established FL frameworks, are sufficient to reconstruct privacy-protected data. Additionally, the attack algorithm is robust to defense strategies that involve adding random noise. We demonstrate the limitations of existing frameworks and propose potential defense strategies analyzing the implications of using differential privacy. The novel insights presented in this study will aid in the improvement of FL frameworks.

**Availability and implementation:**

The code examples are provided at GitHub (https://github.com/manuhuth/Data-Leakage-From-Covariances.git). The CNSIM1 dataset, which we used in the manuscript, is available within the DSData R package (https://github.com/datashield/DSData/tree/main/data).

## 1 Introduction

Large-scale datasets have been shown to be highly valuable for data-driven discovery in various fields, such as clinical research (Harrison *et al.* 2020, Dayan *et al.* 2021, Jannasch *et al.* 2022), self-driving cars (Pokhrel and Choi 2020, Posner *et al.* 2021), and smartphone keyboard word predictions (Chen *et al.* 2019). The COVID-19 pandemic has highlighted the importance of the rapid acquisition of new evidence for interventions in public health. Yet, data are often collected by different sides, e.g. hospitals, and established legal frameworks limit direct sharing (Hansen *et al.* 2021), reducing the speed, and statistical power of the analyses with possibly harmful consequences for patients (Bentzen *et al.* 2021). To facilitate the integrative analysis of distributed datasets, federated learning (FL) has been introduced by Google Researchers in 2016 (McMahan *et al.* 2017). This supposedly allows for privacy-preserving estimation of statistical models from distributed data, making it an essential tool for the rapid assessment of new treatments to improve the fast acquisition of evidence-based interventions in public health. Security is a key topic in the field, as data leakage can result in deontological and consequentialist privacy harms (Price and Cohen 2019).

**Figure 1.**
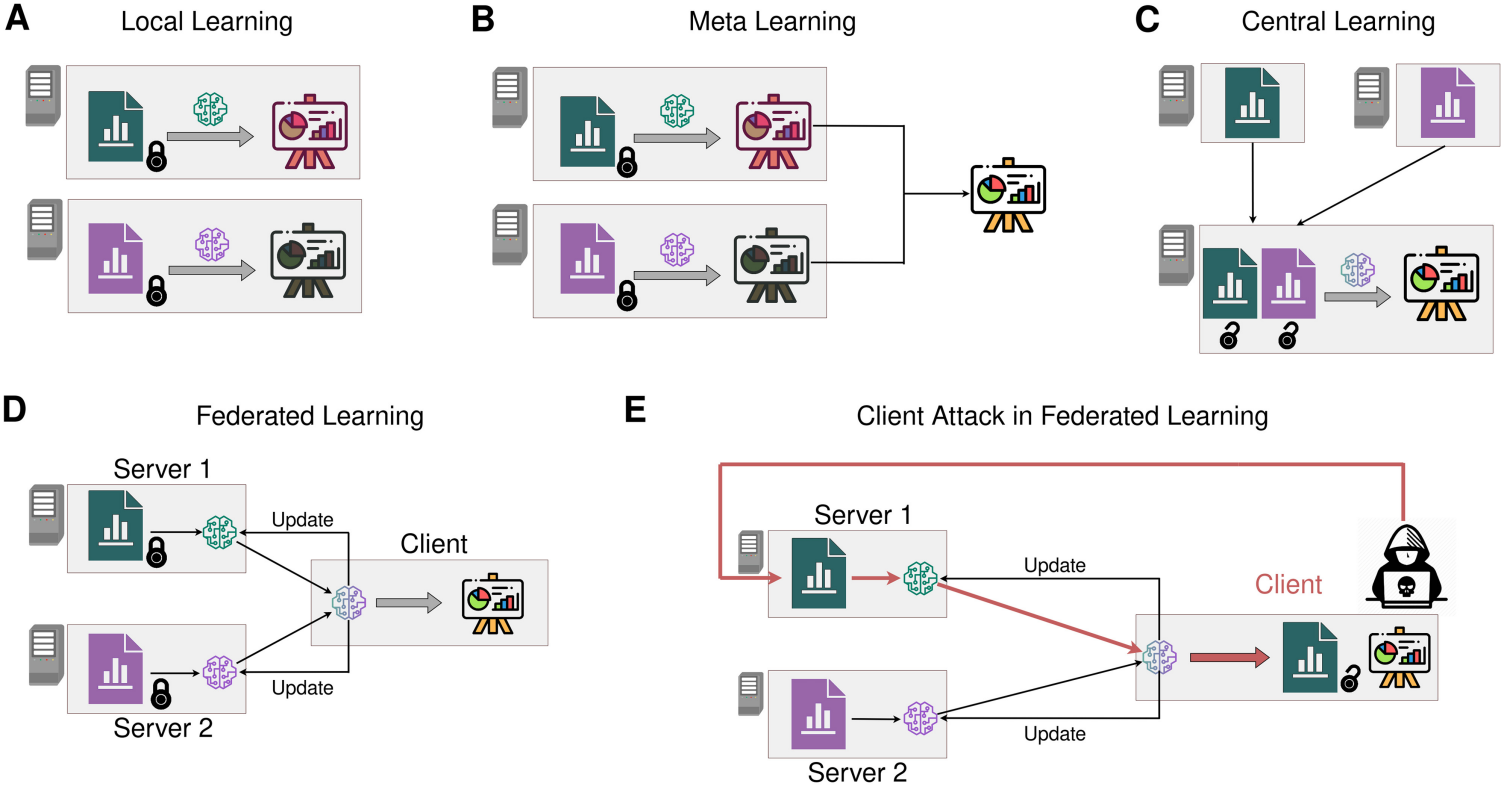
Concept of (attacks in) FL. (A) In Local Learning, all models are trained separately on different servers. (B) In Meta Learning, all models are trained separately on different servers but individual results are subsequently averaged to obtain meta results. (C) In Central Learning, the data are pooled and one model is trained. Hence, the data must be shared. (D) In FL, the data are kept private on the servers. One model is trained with continuous updates between the client and the servers. (E) Illustration of a client-side attack in FL. A malicious client uses the information received from the server to retrieve private data. This figure has been designed using resources from Flaticon.com.

FL is based on sharing informative summary statistics by individual data owners (each running a data server) with a central hub (Fig. 1D). This central hub is responsible for model building. Servers do not share individuals’ data but only non-disclosive summary statistics. This approach is considered privacy-preserving. The focus on privacy in these areas has naturally precipitated extensive research on potential attack vectors. In particular, that sharing parameter gradients—a particular type of summary statistic—in deep neural structures can reveal the training data (Zhu *et al.* 2019, Geiping *et al.* 2020, Huang *et al.* 2021, Yin *et al.* 2021). Algorithms were able to recreate images and texts (Zhu *et al.* 2019). Further, data leakage threats have been summarized (Jere *et al.*, 2021).

**Figure 2.**
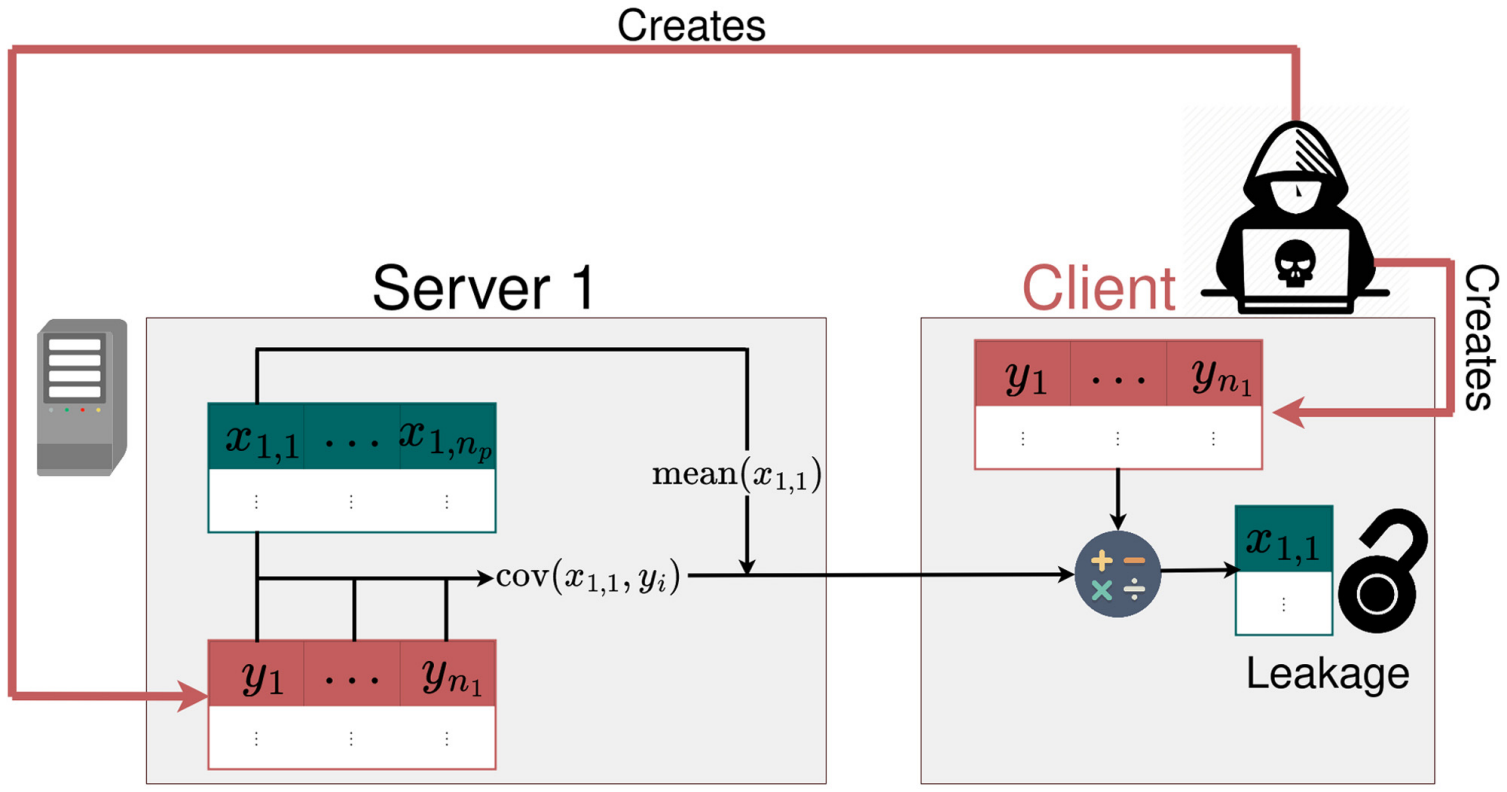
Covariance-Based Attack Algorithm setup for reconstructing data $x_{1,1}$ on the first server. The malicious client generates linear independent vectors $y_{1},\ldots ,y_{n_{1}}$ on the server and client side, computes the covariances of them together with the attacked vector $x_{1,1}$, and returns them with the mean of $x_{1,1}$ to the client side. Subsequently, the returned information is used with the means of $y_{1},\ldots ,y_{n_{1}}$ to compute $x_{1,1}$ on the client side. The algorithm can be repeated for all $x_{j,k}$ to obtain the full dataset. This figure has been designed using resources from Flaticon.com.

In this study, we complement the previous work by focusing on attacks based on basic functionalities that are available in established FL frameworks. We consider the possibilities of a malicious client who tries to obtain the data stored across different data owners and introduce a new attack concept. In principle, it is worth noting that the malicious client could also be a data owner acting as a client. To perform the attack, we generate known linearly independent vectors on each server. After concatenating them on the client side, we use sample means and sample covariances to reconstruct the server-side data nearly up to numerical precision (Fig. 2). In contrast to the well-studied gradient approach, the presented method requires comparatively little time, and no model knowledge. Moreover, our algorithm carries desirable statistical properties: repeated execution allows for exact data reconstruction even if random additive noise is applied to the covariance and means. In our opinion, this combination of features makes this attack strategy more problematic than any approach outlined previously. We discuss our algorithm theoretically and demonstrate its use in the open-source frameworks DataSHIELD (version 6.2.0) (Marcon *et al.* 2021), TensorFlow Federated (version 0.36.0) (Abadi *et al.* 2015) and implemented a proof-of-concept for further platforms using PyTorch (Paszke *et al.* 2019).

## 2 Materials and methods

### 2.1 Covariance-Based Attack Algorithm

**Algorithm 1** Covariance-Based Attack Algorithm

 **Input:** Position of attacked server side variable $x_{j,k}$

 **Output:** Retrieved data $x_{j,k}$

**Require:** Data matrix $X_{j}$ on the server side, function Mean(x) returning the sample mean, function $\text{Cov}(x,x^{\prime })$ returning the sample covariance, algorithm $A(n_{j})$ returning $n_{j}$ known linearly independent vectors $y_{i}\in R^{n_{j}}$ on the server side and their column-wise collection as a matrix *Y* on the client side

1: **procedure**

2:  $Y,y_{1},\ldots ,y_{n_{j}}\leftarrow A(n_{j})$       ▹ Client and server side

3:  initialize $\tilde{V},\tilde{m}\in R^{n_{j}}$              ▹ Client side

4:  **for** *i* in $1:n_{j}$**do**

5:   $\tilde{m}[i]\leftarrow \text{Mean}(y_{i})$              ▹ Client side

6:   $\tilde{V}[i]\leftarrow \text{Cov}(x_{j,k},y_{i})$             ▹ Client side

7:  **end for**

8:  $x\leftarrow (n_{j}-1){\left(Y^{T}\right)}^{-1}\tilde{V}+n_{j}\;\text{Mean}(x_{j,k}){\left(Y^{T}\right)}^{-1}\tilde{m}$▹ Client side

9: **return** *x*

10: **end procedure**

The distributed infrastructure consists of $n_{h}$ servers. The *j*-th server hosts observations $s=1,\ldots ,n_{j}$ (e.g. patient datasets), each with information for variables $k=1,\ldots ,n_{p}$. Accordingly, each server stores a data matrix $X_{j}=\left(\begin{array}{ccccc} x_{j,1} & \ldots & x_{j,k} & \ldots & x_{j,n_{p}} \end{array}\right)\;\text{with}\;x_{j,k}\in R^{n_{j}},$ where each vector $x_{j,k}$ contains information about the variable *k* for all $n_{j}$ samples on the server *j*. Without loss of generality, the malicious client focuses on a specific variable *k* on a specific server *j*, denoted by $x_{j,k}$. Retrieving the remaining variables and servers can be subsequently obtained analogously. We assume that the attacker has at least three basic tools: (T1) a sample mean function Mean(x), (T2) a sample covariance function Cov(x,y), and (T3) an algorithm A generating $n_{j}$ linearly independent vectors $y_{i}\in R^{n_{j}}$ on the server side and their column-wise collection as a matrix $Y\in R^{n_{j}\times n_{j}}$ on the client side, $A(n_{j})=\left(\begin{array}{ccccc} y_{1} & \ldots & y_{i} & \ldots & y_{n_{j}} \end{array}\right)=Y\;\text{with}\;y_{i}\in R^{n_{j}}$ (Fig. 2).

These requirements are met by many distributed analysis frameworks, virtually all of which include functions for computing sample means (T1) and covariances (T2). The input of the covariance function is usually not restricted to subsets of the data matrix *X* but allows for other inputs *y* (T2). The availability of a function for the construction and sharing of linearly independent vectors (T3) might seem the least obvious, but it is available in most tools. For instance, it is necessary in the context of optimization via federated averaging: The client receives the server-side gradients, updates the parameters, and sends them back to the servers. Therefore, for any system where this operation is possible, assumption (T3) must be satisfied, since we need to be able to send vectors from the client side to the server side. Well-known and widely used distributed analysis frameworks for which assumptions (T1)–(T3) are fulfilled are TensorFlow Federated (Abadi *et al.* 2015) and DataSHIELD (Marcon *et al.* 2021).

Assuming that (T1)–(T3) are met, the centerpiece of our algorithm is the fact that evaluating the sample covariance makes it possible to reconstruct the inner vector products between the attacked vector $x_{j,k}$ and the linearly independent vectors $y_{1},\ldots ,y_{n_{j}}$. Computing all covariances between $x_{j,k}$ and the $n_{j}$ linearly independent vectors yields $n_{j}$ equations

(1) $$\begin{array}{c} \begin{array}{cc} \text{Cov}\left(x_{j,k},y_{i}\right) & =\frac{1}{n_{j}-1}\;y_{i}^{T}x_{j,k} \end{array}\\ \;\;\;\;\;\;\;-\frac{n_{j}}{n_{j}-1}\;\text{Mean}(x_{j,k})\;\text{Mean}(y_{i}). \end{array}$$

This linear system of equations can be solved for $x_{j,k}$ and written in matrix form as

(2) $$\begin{array}{c} \begin{array}{cc} x_{j,k} & =(n_{j}-1)\cdot {\left(Y^{T}\right)}^{-1}\underset{:=\tilde{V}}{\underset{︸}{\left(\begin{array}{c} \text{Cov}\left(x_{j,k},y_{1}\right)\\ \vdots \\ \text{Cov}\left(x_{j,k},y_{n_{j}}\right) \end{array}\right)}} \end{array}\\ \;\;\;\;+n_{j}\cdot \text{Mean}(x_{j,k})\cdot {\left(Y^{T}\right)}^{-1}\underset{:=\tilde{m}}{\underset{︸}{\left(\begin{array}{c} \text{Mean}(y_{1})\\ \vdots \\ \text{Mean}(y_{n_{j}}) \end{array}\right)}}, \end{array}$$

where the right-hand side of (2) is known by the malicious client. Derivations of the computations are reported in the Supplementary Appendix. The presented procedure can be repeated for each variable $k=1,2,\ldots ,n_{p}$ and server $j=1,2,\ldots ,n_{S}$, using the same or newly generated linearly independent vectors $y_{i}$, until all data $X_{1},\ldots ,X_{n_{s}}$ are obtained.

As the covariance calculation is essential, we refer to the strategy as “Covariance-Based Attack Algorithm.”

### 2.2 Computation complexity of data reconstruction grows linearly with sample size

To study the applicability of the Covariance-Based Attack Algorithm, we considered the scaling of the computation time with growing sample size $n_{j}$. As computation time, we consider the wall time required to obtain the result.

In theory, the sample size determines the time requirements in different ways. First, it determines the size of the system of Equations (2). This size is identical to $n_{j}$, meaning that $n_{j}$ requests must be sent to the *j*-th server. The communication overhead for a request is constant, but the computation time will generally grow linearly with $n_{j}\;\left[O(n_{j})\right]$, as the dimensionality of the scalar product increases. Secondly, the computation time for solving the linear system from (2) grows cubically $\left[O(n_{j}^{3})\right]$ using the solve command in R and the linalg.inv command in Python available through the NumPy library. Hence, there are linear, and cubic contributions, with different pre-factors, to the computation time.

**Figure 3.**
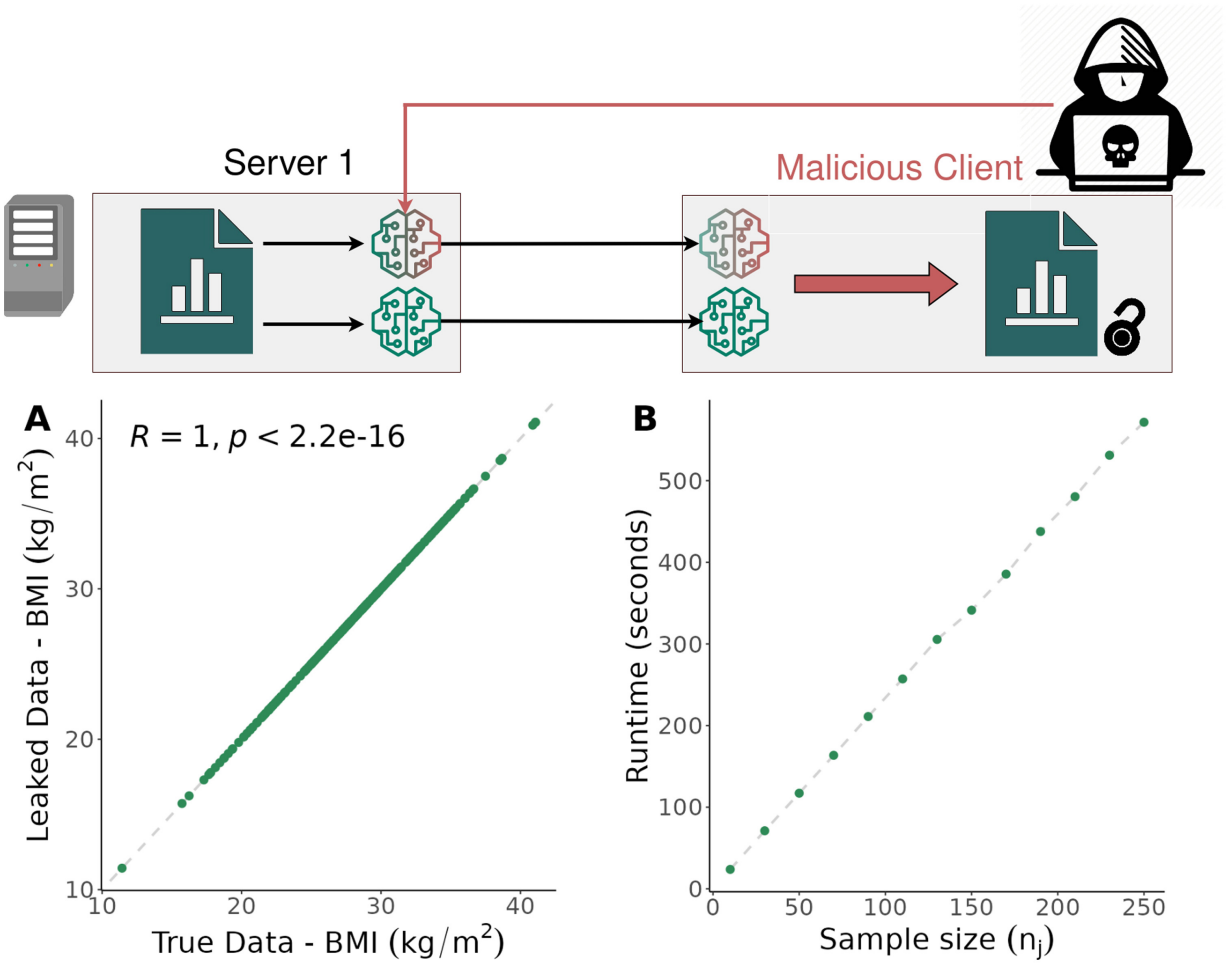
Leakage results and computation times for DataSHIELD. (A) True data values from the first server of the CNSIM dataset versus the corresponding retrieved data provided by the Covariance-Based Attack Algorithm. (B) Computation time of the algorithm for different sample size. This figure has been designed using resources from Flaticon.com.

To evaluate the scaling behavior in practice, we considered subsets of the CNSIM dataset of different sizes and determined the wall time required to complete the attack (Fig. 3B). The CNSIM dataset from the DataSHIELD tutorial (Westerberg and Wilson 2022) (https://data2knowledge.atlassian.net/wiki/spaces/DSDEV/pages/931069953) to ensure an easy-to-reproduce test case. This dataset consists of three servers with a total of 9379 synthetic observations of 11 personalized obesity-related variables. We have reconstructed information on individual body mass index (BMI) measurements from the first server using a complete case analysis with a sample size of $n_{j}=250$.

We observed linear scaling (Fig. 3B), meaning that the communication overhead determines overall wall time. Indeed, even for the largest dataset considered, matrix inversion required only 0.004 s, meaning that it contributed only $7\times 10^{-6}$ percent to the overall time.

The essentially linear scaling behavior in the relevant regime, compared to the theoretically cubic scaling behavior, leads to this attack being feasible in many real-world scenarios. However, if the sample size becomes very large the cubic term will eventually dominate the run time. A possibility of tackling this bottleneck would be to use Krylov-subspace methods (Sogabe 2022), which can provide approximate solutions for the matrix inverse with controllable accuracy and computational cost.

### 2.3 Established FL platforms are vulnerable to the algorithm

#### 2.3.1 DataSHIELD is vulnerable to the algorithm

To demonstrate the Covariance-Based Attack Algorithm and the vulnerability of existing distributed analysis frameworks, we considered different software packages. First, we provide an example implementation in DataSHIELD (version 6.2.0) and its base package dsBaseClient (Marcon *et al.* 2021). This tool is well established and used in various biomedical applications (Pastorino *et al.* 2021, Pearce *et al.* 2021, Peñalvo *et al.* 2021, Jannasch *et al.* 2022, Moira *et al.* 2022) in which data sharing is limited, e.g. to ensure compliance with privacy regulations, such as the General Data Protection Regulation (GDPR).

In DataSHIELD, analysts are confined to pre-defined functions approved by data owners, limiting the risk of attackers introducing malicious code. However, data owners lack the ability to review the code before potential attacks occur.

DataSHIELD meets the requirements (T1)–(T3) and is therefore vulnerable to the Covariance-Based Attack Algorithm. The functions to compute sample means (T1) and sample covariances (T2) are ds.mean and ds.cov, respectively. These functions return the means and covariances directly, but require mild conditions on the attacked data $x_{j,k}$: (C1) the sample sizes $n_{j}$ must exceed the thresholds $n_{j}>3$ (ds.mean) and $n_{j}>6$ (ds.cov); and (C2) both levels of a dichotomous variable must occur at least three times in the given data vectors. The conditions (C1) and (C2) ensure a privacy-preserving analysis if the functions are applied once. If any of the assumptions (C1) and (C2) were violated, descriptive statistics or further analysis with $x_{j,k}$ would be impossible. Therefore, it is reasonable to assume their validity. In our example, the data have $n_{j}=250$ observations of a continuous variable so that requirements (C1) and (C2) are clearly satisfied. The construction of $n_{j}$ linearly independent vectors (T3) can be implemented in several ways. We used the function ds.dmtC2S to send client side matrices to the server side. Hence, it is possible for the client to create suitable linearly independent vectors $y_{i}$ on the client side and to send them to the server side. Note that since the covariance operation is performed on $x_{j,k}$ and all $y_{i}$, (C1) and (C2) must hold for all $y_{i}$ as well. Since $x_{j,k}$ and $y_{i}$ have the same length $n_{j}$, (C1) holds. To meet (C2) and the linear independence condition, we draw each element $y_{i}$ from a standard normal distribution, so that $y_{i}$ almost surely consists of $n_{j}$ distinct entries and that $y_{1},\ldots ,y_{n_{j}}$ are almost surely linearly independent. In principle, however, the malicious client can use any linearly independent vectors $y_{1},\ldots ,y_{n_{j}}$ that meet the requirements (C1) and (C2).

After creating all linearly independent $y_{i}$ on the server and client side and computing the relevant means and covariances, the data can be obtained as described in (2). Our evaluation of the above example shows that the true data can be reconstructed almost perfectly (see Fig. 3A). The Pearson correlation coefficient between true and retrieved BMI values is 1.0. The highest absolute error observed is $2\times 10^{-12}$, which is close to numerical accuracy. This demonstrates that the Covariance-Based Attack Algorithm is not limited to theoretical settings. Instead, data leakage can also be achieved in real-life setups.

#### 2.3.2 The Covariance-Based Attack Algorithm is robust against noise perturbations in DataSHIELD

The Covariance-Based Attack Algorithm allows for the reconstruction of the data on the servers. We further investigated whether our approach is robust to adding zero-mean noise to the means and covariances before returning them to the client. In this case, the client observes noise-corrupted data estimates

$$\begin{array}{c} x_{j,k}^{\mathrm{noisy}}=x_{j,k}+(n_{j}-1){\left(Y^{T}\right)}^{-1}\varepsilon +n_{j}{\left(Y^{T}\right)}^{-1}\tilde{m}\gamma , \end{array}$$

with zero-mean and finite-variance noise terms ε and γ.

**Figure 4.**
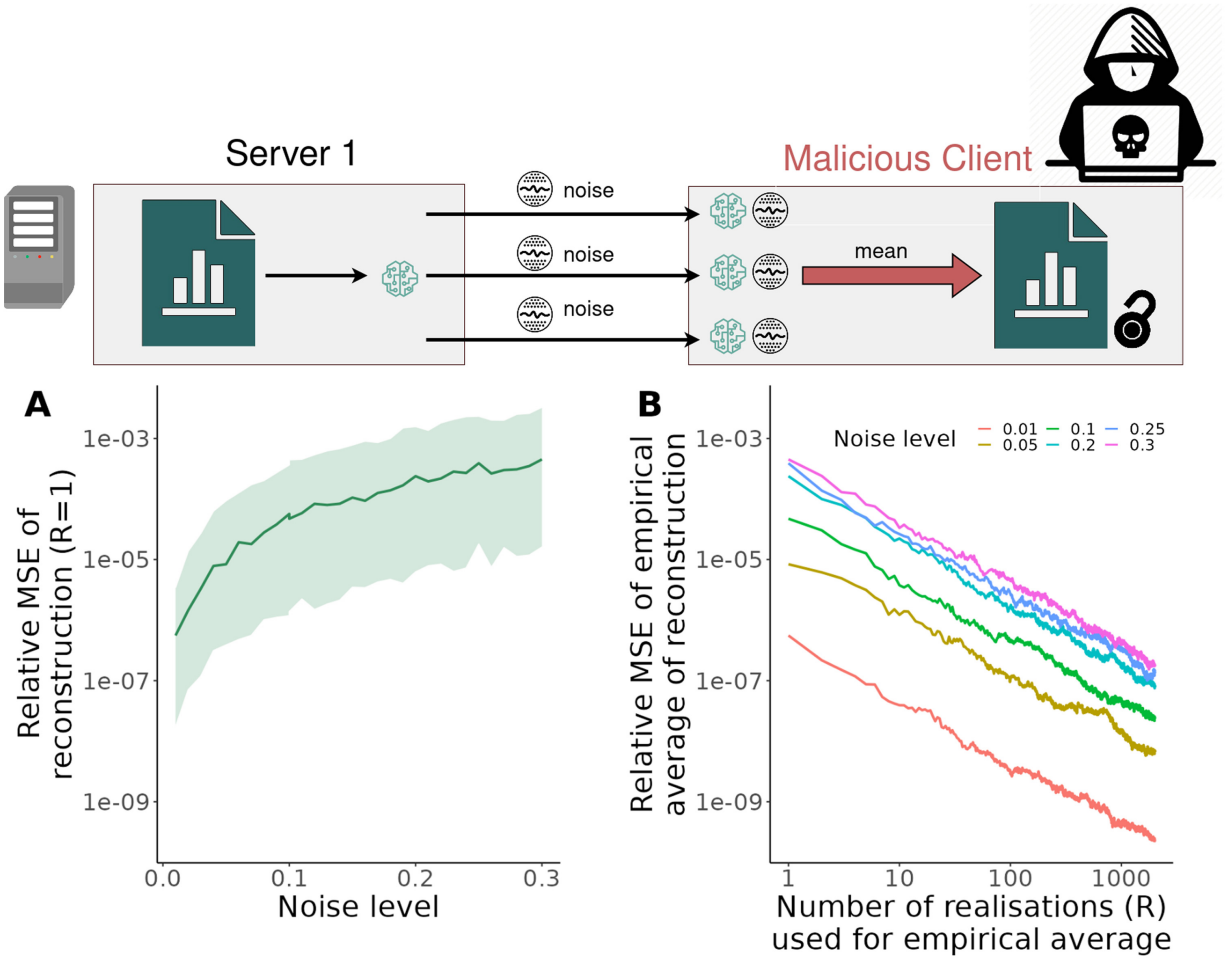
Robustness of Covariance-Based Attack Algorithm to normally distributed noise on means and covariances. (A) Relative mean squared error (MSE) of the reconstructed data values for different noise level if only a single realization is available (R=1). The median (line) and the 5th–95th-percentile (area) of 200 replicates are depicted. (B) MSE of the empirical mean of reconstructed data values obtained from different numbers of realization (R=1,…,1000) and four different noise levels. The median (line) of 200 replicates is depicted. This figure has been designed using resources from Flaticon.com.

The noise-corrupted data estimate $x_{j,k}^{\mathrm{noisy}}$ can be decomposed into the true data $x_{j,k}$ and a noise component so that the malicious client cannot retrieve the original data (Fig. 4A). However, the malicious client is, given suitable communication and computational budgets, which is given e.g. in DataSHIELD, able to run the algorithm *R* times. If *R* is sufficiently large, the zero-mean noise components average out such that the mean $\frac{1}{R}\sum_{r=1}^{R}x_{j,k,r}^{\mathrm{noisy}}$ converges in probability with rate $\sqrt{R}$ to the data $x_{j,k}$ (Fig. 4B). We provide a proof of the convergence rate in the Supplementary Appendix. Hence, even if noise is added to the means and covariances, a malicious client can retrieve the data if no further restrictions on the analyst are imposed.

#### 2.3.3 TensorFlow federated is vulnerable to the algorithm

To assess whether other tools allow for the implementation of similar attack strategies, we considered TensorFlow Federated (version 0.36.0) (Abadi *et al.* 2015). In contrast to DataSHIELD, this open-source framework for computations on decentralized data is meant for experimentation with FL. Users can define their own functions, which must be reviewed in a real-world setting. Yet, if experimentation environments allow for (non-trivial) disclosive computations, these are likely to find their way into application.

Accordingly, we evaluated the possibility of implementing the Covariance-Based Attack Algorithm using a set of basic functions. (Note that the developers of TensorFlow Federated use the terms client and server in a way opposite to that of the DataSHIELD community. To avoid confusing the reader, we stick to the convention of DataSHIELD, with the client being the central hub and the servers being the data owners).

Our assessment revealed that TensorFlow Federated meets the tool requirements (T1)–(T3) and allows for the implementation of the Covariance-Based Attack Algorithm. Functions can be constructed in TensorFlow Federated by wrapping functionalities from Python packages, e.g. TensorFlow or numpy, in a function and labeling it with tf_computation. To compute sample means (T1), a function that computes the average of $x_{j,k}$, e.g. using numpy.mean, can be implemented. For (T2), one can for instance wrap the function stats.covariance from the TensorFlow probability package. Both functions need to be applied with the functionality of federated_map to return values from the server side. Since TensorFlow Federated does not enforce further privacy leakage checks, these functions do not have requirements that are equivalent to (C1) and (C2) for DataSHIELD. However, we expect that if TensorFlow Federated is used in real-world applications, further disclosiveness checks, similar to (C1) and (C2), will be implemented. For (T3), TensorFlow Federated offers the tff.federated_broadcast function, which is similar to the function ds.dmtC2S as it sends objects from the client to the server side. Due to the current lack of requirements, such as (C1) and (C2), the vectors $y_{1},\ldots ,y_{n_{j}}$ must be linearly independent but no further restrictions have to be imposed.

The implementation of the Covariance-Based Attack Algorithm in TensorFlow Federated was applied to the aforementioned CNSIM dataset. We found that this allows for a reconstruction of the data up to numerical accuracy (Supplementary Fig. S6). Hence, data leakage is also possible in TensorFlow Federated, using algorithms that appear to be non-disclosive. This raises questions regarding the suitability of the framework for experimentation with FL.

#### 2.3.4 PyTorch-based platforms used in real-world applications are vulnerable to the algorithm

Further FL tools, such as Nvidia Flare (Roth *et al.* 2022), IBM FL (Ludwig *et al.* 2020), Intel’s OpenFL (Foley *et al.* 2022), and PySyft (Ziller *et al.* 2021), are widely used in real-world applications that handle sensitive data. For example, Nvidia Flare is used by Microsoft Azure, American College of Radiology, Rhino Health, and others as indicated on their website (https://developer.nvidia.com/flare). These tools advertise their flexibility in using popular Python-based deep learning packages like PyTorch (Paszke *et al.* 2019). As for TensorFlow Federated, users can define their own functions which must be reviewed by data security experts. In light of the potential for malicious implementation of algorithms, we evaluated the feasibility of implementing the Covariance-Based Attack Algorithm on these platforms.

Our analysis demonstrated that the platforms under examination fulfill the necessary tool requirements (T1)–(T3), enabling the implementation of a Covariance-Based Attack algorithm. Users of these platforms have the ability to specify their models in PyTorch, Tensorflow, or Numpy, and the platforms subsequently integrate the specified models into a FL workflow so that the final model specification is ultimately determined by the client. To compute sample means (T1) one can apply torch.mean on the variables of interest. Meeting the tool requirement (T3) of generating linearly independent vectors directly is not possible. However, it is possible to jointly implement (T3) and (T2) by using a linear model $M_{i}$ for each random vector $y_{i}$, specified in PyTorch. In this model, the dependent variable is hard-coded to be $y_{i}$, resulting in the following equation

(3) $$\begin{array}{c} x_{j,k}=y_{i}\beta +\varepsilon \end{array}.$$

The estimate $\hat{\beta }=\frac{\text{Cov}(x_{j,k},y_{i})}{\text{Var}(y_{i})}$ is returned to the analyst. The analyst can then compute the corresponding covariance on the client side since $\text{Var}(y_{i})$ is known. By using this method, both requirements (T2) and (T3) can be met.

The examined platforms do not enforce (C1) and (C2) as built-in security measures. However, additional security features are implemented, e.g. models must undergo a review process prior to being utilized with real-world data. Our work can improve this process by raising the awareness to potential disclosive models. Moreover, the execution of algorithms might be limited by a privacy budget in accordance with differential privacy principles, as outlined in Dwork and Roth (2014).

#### 2.3.5 Differential privacy limits the algorithms use but precludes standard analysis

Our proposed algorithm, the Covariance-Based Attack, demonstrates effective data retrieval from platforms that do not implement computational privacy budgets. To assess the impact of such budgets on our method, we evaluated its robustness by testing its performance when platforms enforce differential privacy restrictions on the client’s analysis. Differential privacy improves security by adding appropriate noise to the output of an algorithm, A, when applied to a dataset, D, in order to ensure that no individual in D can be directly identified (Dwork and Roth 2014). The privacy budget, $\epsilon_{A}$, which controls the level of noise added, is allocated to the analyst and must be used with caution, as it limits the number of algorithm calls that can be made by the analyst. It has shown its applicability for large deep learning models (Abadi *et al.* 2016) but its use to standard statistical tools, in which particularly linear models consume a large proportion of the privacy budget, has not been studied extensively.

The privacy budget consumed by the Covariance-Based Attack algorithm, denoted as $\epsilon_{\text{att}}$, can be upper-bounded by the sum of the individual privacy budgets allocated for the computation of covariances and means

(4) $$\begin{array}{c} \epsilon_{\text{att}}=\sum_{i=1}^{n_{j}}\epsilon_{\text{cov}(x_{j,k},y_{i})}+\epsilon_{\text{mean}(x_{j,k})}. \end{array}$$

Detailed mathematical descriptions of the privacy budget consumption of the mean $\epsilon_{\text{mean}(x_{j,k})}$ and covariances $\epsilon_{\text{cov}(x_{j,k},y_{i})}$ are given in the Supplementary Appendix.

**Figure 5.**
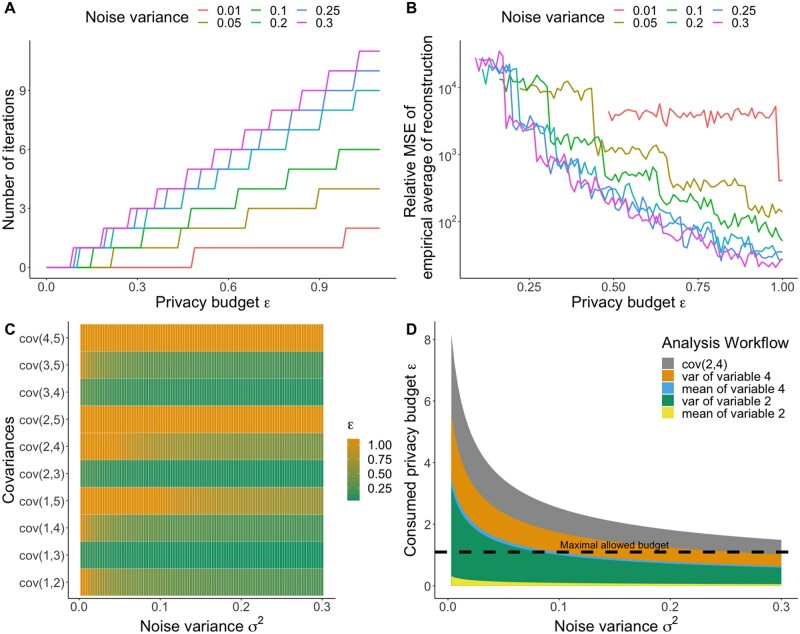
(A) Number of possible iterations of the Covariance-Based Attack Algorithm for different noise levels still satisfying ϵ-differential privacy using the CNSIM dataset. (B) Error of the Covariance-Based Attack Algorithm after allowed numbers of iteration for different noise levels still satisfying ϵ-differential privacy using the CNSIM dataset. The median (line) of 200 replicates is depicted. (C) Privacy budget consumed by computing the covariances between metric variables in the CNSIM dataset for different noise levels. The consumed budget ϵ is truncated at the maximal allowed privacy budget ln(3). (D) The privacy budget consumed by performing a standard analysis on two variables (computing the mean and variance of two metric variables and their covariance).

By specifying a privacy budget, data owners face the trade-off between more function calls or increasing security. More function calls could result in more informative outcomes but increases the risk of data leakage, whereas less function calls increase security but could limit the statistical results. We examined this trade-off using the CNSIM dataset. First, we computed the possible number of runs of the Covariance-Based Attack Algorithm depending on the privacy budget, and examined how the relative error of reconstruction changes with the privacy budget (Fig. 5A and B). We varied the privacy budget parameter, ϵ, between ln(1.01) and ln(3), based on the range defined in Dwork and Roth (2014). Second, we computed covariances (Fig. 5C) between variables of the CNSIM data to see how much privacy budget is consumed by standard descriptive statistics. Third, we analyzed the consumption of the privacy budget for a basic analysis workflow with means, and (Co-)Variances of two variables (Fig. 5D) examining if a standard analysis is still feasible under the restrictions of differential privacy.

Increasing the noise variance of the outputs of the covariances and means enables the analyst to perform more iterations of the Covariance-Based Attack algorithm, as shown in Fig. 5A. However, even at the highest noise variance, it is not possible to obtain a sufficient number of runs of the algorithm to recover the true data by averaging out the noise, as demonstrated in Fig. 5B. Interestingly, more noise leads in general to a higher accuracy of the algorithm for the same privacy budget ϵ since more iterations of the algorithm can compensate increasing noise as one can see in Fig. 5B.

However, the allocated privacy budgets are not sufficient for a non-malicious analyst to perform covariance computations on the five metric variables of the CNSIM dataset. Even when using high noise variances, the computation of one covariance consumes already the whole privacy budget, as shown in Fig. 5C. The standard analysis workflow could not be computed for all considered noise variances due to the consumed accumulated privacy budget, as illustrated in Fig. 5D.

## 3 Discussion

FL has demonstrated its utility and importance in various fields, and its significance has been further highlighted during the SARS-CoV-2 pandemic, with many consortia utilizing it extensively (Dayan *et al.* 2021, Warnat-Herresthal *et al.* 2021, Tacconelli *et al.* 2022). However, it is crucial to ensure that the data of the participating servers remains protected. To accomplish this, a thorough examination of attack strategies is necessary. In this study, we introduce the novel Covariance-Based Attack Algorithm, which highlights the vulnerability of established FL systems.

We have demonstrated that a malicious client could leverage the Covariance-Based Attack Algorithm to extract data from a FL system. Our approach differs from previous studies that focused on information leakage through gradients obtained from deep neural models. It relies on constructing linearly independent vectors on the server side and utilizing sample means and sample covariance functions that are accessible to the client. The attack approach is efficient, scalable, and can be easily implemented, and it is not hindered by noise perturbations if the number of function calls is not restricted. However, implementing differential privacy can prevent the algorithm but would also hinder the application of (linear) analysis tools making differential privacy impractical to use if rather standard tools like linear models should be used. Our algorithm has been successfully implemented on DataSHIELD (version 6.2.0) and TensorFlow Federated (version 0.36.0), where we were able to reconstruct the data. Furthermore, we have implemented a proof-of-concept using PyTorch (version 1.13.0) showing the usability of the algorithm on other FL platforms. The impact of our work on different platforms can be classified based on their functionality. For platforms, such as DataSHIELD, which provide pre-written functions and allow unlimited queries, our research aids platform designers in scrutinizing packages to ensure the highest security standards. Conversely, platforms that enable users to write their own code face the challenge of potential attack infiltration. In this context, our work enhances code review processes by raising awareness of attacks, including the one proposed in our study.

The Covariance-Based Attack is a notable example of attack algorithms within a class that utilize invertible projection matrices applied to known (random) projections. In essence, any sequence of queries enabling the computation of such a projection matrix can potentially lead to data leakage, as observed in the case of the Covariance-Based Attack Algorithm. This presents a significant challenge for platform developers, as these hidden projections may be embedded within multiple queries, as demonstrated by the Covariance-Based Algorithm.

Our algorithm illustrates an attack possibility using linear mappings. We would also like to emphasize that while our algorithm focuses on the linear case, the broader implications apply to non-linear models as well, as shown for the sharing of large-scale gradient information (Zhu *et al.* 2019). Although the computations may be less straightforward in non-linear cases, the underlying risks and privacy concerns persist. Thus, it is imperative to consider any form of extensive summary statistics as potential threats until their impact is thoroughly investigated and verified.

Our findings reveal that the existing functionalities of FL frameworks need to be scrutinized in regard to data leakage threats. It is important to note that the proposed strategy can only be executed if the attacker can act as the client, which typically requires obtaining log-in credentials. However, even with this access, the security of the data should not solely rely on the trustworthiness of the client, as the Covariance-Based Attack Algorithm demonstrates that data can still be retrieved. The need for FL would be eliminated if the security of the data is dependent on the trustworthiness of the client alone, as it could be replaced by Centralized Learning. Furthermore, this raises concerns about compliance with the GDPR and about responsibility and liability in the event of unknown attack strategies.

Differential privacy has shown to limit the applicability of the algorithm, however, also precludes analysis for standard statistical tools. To address the vulnerability for platforms with the focus on standard statistical models, as DataSHIELD, we propose to tackle the issue induced by (T3). An effective solution would be to immediately process these vectors within a function instead of creating a vector on the server side avoiding the possibility of computations with client-known objects. Furthermore, functions that allow for the creation of objects on the client side need to be screened with respect to the trade-off between functionality and data leakage threats. After getting in touch with the developers of DataSHIELD, we have jointly examined which functions yield potential data leakage risks. These functions will be blocked by default in the next update (DataSHIELD 6.4), which is currently under development.

With this work, we contribute to the growing literature on data leakage problems in FL systems and aim to support research on Federated Algorithms and raise awareness about potential security risks. We anticipate that our results will play a role in developing design criteria for the architecture of FL platforms. We have shown that existing systems need to be enhanced to minimize the risk of data leaks.

## Supplementary Material

btad531_Supplementary_Data
