# Supplementary material for: Accessibility of covariance information creates vulnerability in Federated Learning frameworks
Source: Bioinformatics. 2023 Aug 30;39(9):btad531. doi: 10.1093/bioinformatics/btad531 (PMC10516515; doi:10.1093/bioinformatics/btad531)
Supplement: btad531_Supplementary_Data [file btad531_supplementary_data.pdf]

Supplementary Material

Proof of correctness for the Covariance-Based Attack Algorithm

In this study, we consider an attack by a malicious client and provide an algorithm for data reconstruction based on covariance information. In the following, we provide the mathematical derivation of the algorithm.

For the attack, it is necessary to compute sample means

$$\text{Mean}(x_{j,k}) = \frac{1}{n_j}\sum_{s=1}^{n_j} x_{j,k}^{(s)},$$

$$\text{Mean}(y_i) = \frac{1}{n_j}\sum_{s=1}^{n_j} y_i^{(s)},$$

and sample covariances,

$$\begin{aligned}\text{Cov}\left(x_{j,k}, y_i\right) =& \frac{1}{n_j - 1}\sum_{s=1}^{n_j}\left(x_{j,k}^{(s)} - \text{Mean}(x_{j,k})\right)\left(y_i^{(s)} - \text{Mean}(y_i)\right)\\ =& \frac{1}{n_j-1}\ y_i^T x_{j,k} - \frac{n_j}{n_j-1}\ \text{Mean}(x_{j,k})\ \text{Mean}(y_i),\end{aligned}$$

for all $i = 1, 2, \ldots, n_j$ on the server side and return them to the client side. The vectors y_i are chosen in a way to ensure their linear independence.

To reconstruct $x_{j,k}$, we exploit the fact that the sample covariances can be reformulated to determine the inner product $y_i^T x_{j,k}$,

$$y_i^T x_{j,k} = (n_j - 1)\ \text{Cov}\left(x_{j,k}, y_i\right) + n_j\ \text{Mean}(x_{j,k})\ \text{Mean}(y_i). \tag{5}$$

We combine the equations for $i = 1, \ldots, n_j$ from (5) to a system of equations in matrix form:

$$\begin{aligned}\underbrace{\begin{pmatrix} y_1^T \\ \vdots \\ y_{n_j}^T\end{pmatrix}}_{Y^T} x_{j,k} =& (n_j - 1)\underbrace{\begin{pmatrix}\text{Cov}\left(x_{j,k}, y_1\right) \\ \vdots \\ \text{Cov}\left(x_{j,k}, y_{n_j}\right)\end{pmatrix}}_{=\tilde{V}_{j,k}}\\ &+ n_j\ \text{Mean}(x_{j,k})\underbrace{\begin{pmatrix}\text{Mean}(y_1)\\ \vdots \\ \text{Mean}(y_{n_j})\end{pmatrix}}_{=\tilde{m}}\\ \implies Y^T x_{j,k} =& (n_j - 1)\ \tilde{V}_{j,k} + n_j\ \text{Mean}(x_{j,k})\ \tilde{m}.\end{aligned}$$

Since the vectors y_i were chosen to be linearly independent, Y, and therefore also Y^T, are invertible. Hence, we can multiply both sides of equation (6) by the inverse of Y^T to obtain

$$x_{j,k} = (n_j - 1)\left(Y^T\right)^{-1}\tilde{V} + n_j\left(Y^T\right)^{-1}\text{Mean}(x_{j,k})\ \tilde{m}, \tag{6}$$

where the right-hand side is known to the client. This provides a constructive proof for the recovery of $x_{j,k}$ using the proposed approach.

The same procedure can be repeated for all n_j servers and all n_p variables, yielding comprehensive information about potentially sensitive data on the servers.

Robustness of the Covariance-Based Attack Algorithm to noise perturbations in DataSHIELD

As a defense strategy against malicious clients, we consider the perturbation of mean and covariance with noise but without the additional use of differential privacy. More specifically, we consider the addition of zero-mean noise to means and covariances on the server side before sending them to the client side. Given only access to noisy data, one might assume that the client will not be able to reconstruct $x_{j,k}$ exactly. However, running the attack algorithm multiple times on the same variable and averaging these results yields a random variable that converges in probability to $x_{j,k}$ such that the malicious client is, given an appropriate communication and computational budget, able to retrieve all information about $x_{j,k}$. We prove that the empirical mean of the noisy results of the Covariance-Based Attack Algorithm $\frac{1}{R}\sum_{r=1}^{R} x_{j,k,r}^{noisy}$, with R denoting the number of calls in an attack, converges in probability to $x_{j,k}$, i.e., formally for any $c > 0$

$$\lim_{R\to\infty}\mathbb{P}\left(\left|\left|\underbrace{\frac{1}{R}\sum_{r=1}^{R} x_{j,k,r}^{noisy}}_{=\text{ empirical mean}} - x_{j,k}\right|\right|_2 \geq c\right) = 0, \tag{7}$$

where $x_{j,k,r}^{noisy}$ is the result of the r-th run of the Covariance-Based Attack Algorithm.

Let ε_r be an n_j dimensional random vector with mean $\mathbb{E}(\varepsilon_r) = 0$ and covariance matrix $\mathbb{V}(\varepsilon_r) = \sigma_\varepsilon^2\mathbb{I}_{n_j}$ for which $\sigma_\varepsilon^2 < \infty$. Let γ_r be a random variable with mean $\mathbb{E}(\gamma_r) = 0$ and variance $\mathbb{V}(\gamma_r) = \sigma_\gamma^2 < \infty$. Furthermore, let γ_r and ε_r be uncorrelated so that $\mathbb{E}(\gamma_r\cdot\varepsilon_r) = 0$. The noisy version of equation (6) is given by

$$\begin{aligned}x_{j,k,r}^{noisy} =& (n_j - 1)\left(Y^T\right)^{-1}(\tilde{V} + \varepsilon_r)\\ &+ n_j\left(Y^T\right)^{-1}(\text{Mean}(x_{j,k}) + \gamma_r)\ \tilde{m}\\ =& x_{j,k} + \underbrace{(n_j - 1)\left(Y^T\right)^{-1}}_{:=A}\varepsilon_r + \underbrace{n_j\left(Y^T\right)^{-1}\tilde{m}}_{:=B}\gamma_r,\end{aligned} \tag{8}$$

such that $x_{j,k,r}^{noisy}$ can be decomposed into the true $x_{j,k}$ and a noise term. Combining (7) and (8) shows that (7) is proven if the mean of the noise term converges in probability to zero, such that it is sufficient to show that

$$\lim_{R\to\infty}\mathbb{P}\left(\left|\left|\frac{1}{R}\sum_{r=1}^{R}(A\varepsilon_r + B\gamma_r)\right|\right|_2 \geq c\right) = 0. \tag{9}$$

This can be shown by applying Markov's Inequality

$$\mathbb{P}\left(\left|\left|\frac{1}{R}\sum_{r=1}^{R}(A\varepsilon_r + B\gamma_r)\right|\right|_2 \geq c\right) < \frac{\mathbb{E}\left(\left|\left|\frac{1}{R}\sum_{r=1}^{R}(A\varepsilon_r + B\gamma_r)\right|\right|_2^2\right)}{c^2}. \tag{10}$$

Since (10) holds for all R, it is sufficient to show that the numerator of the right-hand side converges to 0 if $R\to\infty$ in order to prove (9). To facilitate notation, the entries of A^TA are denoted by $a^{(s,s')}$ and the entries of ε_r by $\varepsilon_r^{(s)}$. Note that the following holds:

- $\forall r, m : \mathbb{E}(\gamma_m\varepsilon_r^T A^T B) = \mathbb{E}(\gamma_m\varepsilon_r^T)A^T B = 0$,
- $\forall l \neq m$: by independence of ε_r and ε_m, $\mathbb{E}(\varepsilon_r^T A^T A\varepsilon_m) = \mathbb{E}(\varepsilon_r^T)A^T A\mathbb{E}(\varepsilon_m)) = 0$ and by independence of γ_r and γ_m that $\mathbb{E}(\gamma_r\gamma_m B^T B) = \mathbb{E}(\gamma_r)\mathbb{E}(\gamma_m)B^T B = 0$,
- $\forall r = m : \mathbb{E}(\varepsilon_r^T A^T A\varepsilon_m) = \sum_{s=1}^{n_j}\sum_{s=1}^{n_j}\mathbb{E}(\varepsilon_r^{(s)}\varepsilon_r^{(s')})a^{(s,s')} = \sigma_\varepsilon^2\sum_{s=1}^{n_j} a^{(s,s)}$ and $\mathbb{E}(\gamma_r\gamma_r B^T B) = \mathbb{E}(\gamma_r^2)B^T B = \sigma_\gamma^2 B^T B$.

The numerator of the right-hand side of (10) can therefore be written as

$$\begin{aligned}
&\mathbb{E}\left(\left|\left|\frac{1}{R}\sum_{r=1}^{R}(A\varepsilon_r + B\gamma_r)\right|\right|_2^2\right)\\
=&\mathbb{E}\left(\frac{1}{R^2}\sum_{r=1}^{R}\sum_{m=1}^{R}\left(\varepsilon_r^T A^T A\varepsilon_m + 2\gamma_m\varepsilon_r^T A^T B + \gamma_r\gamma_m B^T B\right)\right)\\
=&\frac{1}{R^2}\left(R\sigma_\varepsilon^2\sum_{s=1}^{n_j} a^{(s,s)} + R\sigma_\gamma^2 B^T B\right)\\
=&\frac{1}{R}\left(\sigma_\varepsilon^2\sum_{s=1}^{n_j} a^{(s,s)} + \sigma_\gamma^2 B^T B\right).
\end{aligned}$$

This is a constant multiplied by R^{-1}. Accordingly, (9) holds and therefore (7) is proven.

To assess the rate of convergence, we can repeat the convergence examinations and multiply the error rate by n_j^α. This yields $\frac{1}{R^{1-\alpha}}\sum_{r=1}^{R}(A\varepsilon_r + B\gamma_r)$. Repeating the previous steps of the Markov Inequality, one obtains

$$\begin{aligned}
&\mathbb{E}\left(\left|\left|\frac{1}{R^{1-\alpha}}\sum_{r=1}^{R}(A\varepsilon_r + B\gamma_r)\right|\right|_2^2\right)\\
=&\mathbb{E}\left(\frac{1}{R^{2-2\alpha}}\sum_{r=1}^{R}\sum_{m=1}^{R}\left(\varepsilon_r^T A^T A\varepsilon_m + 2\gamma_m\varepsilon_r^T A^T B + \gamma_r\gamma_m B^T B\right)\right)\\
=&\frac{1}{R^{2-2\alpha}}\left(R\sigma_\varepsilon^2\sum_{s=1}^{n_j} a^{(s,s)} + R\sigma_\gamma^2 B^T B\right)\\
=&\frac{R}{R^{2-2\alpha}}\left(\underbrace{\sigma_\varepsilon^2\sum_{s=1}^{n_j} a^{(s,s)} + \sigma_\gamma^2 B^T B}_{\text{constant}}\right)\\
=&\frac{1}{R^{1-2\alpha}}\left(\underbrace{\sigma_\varepsilon^2\sum_{s=1}^{n_j} a^{(s,s)} + \sigma_\gamma^2 B^T B}_{\text{constant}}\right)
\end{aligned}$$

From the last line, it follows that the error converges for $\alpha \in (\infty, 0.5)$ and therefore the upper bound for the rate of convergence is 0.5.

In the manuscript, we provide an analysis of the mean squared error for different numbers of calls from an attacker and different levels of noise. The relative mean squared error (RMSE) is here defined as

$$\text{RMSE} = \frac{\left|\left|\frac{1}{R}\sum_{r=1}^{R} x_{j,k,r}^{noisy} - x_{j,k}\right|\right|_2^2}{||x_{j,k}||_2^2}. \tag{11}$$

Derivation of privacy budget consumption of mean and covariances

The privacy budget consumption of the mean (covariance) algorithm is given by

$$\epsilon_{\mathcal{A}}^2 \geq \frac{2\ S_{\mathcal{A}}^2 \log(1.25\ n_j^2)}{\sigma^2},$$

where σ^2 is the noise level of the normally distributed noise that is added to the output of the mean (covariance). $S_{\mathcal{A}}$ is the algorithm dependent local sensitivity which is the maximum amount that the output of a function can change given a change of a single input. It therefore determines the amount of noise that needs to be added to the output of a function in order to ensure that it satisfies the privacy guarantees of differential privacy (Dwork, Roth, et al., 2014) Mathematically, the local sensitivity $S_{\mathcal{A}}$ is defined as

$$\begin{aligned}
S_{\mathcal{A}} = \max_{\mathcal{D}}|\mathcal{A}(\mathcal{D}) - \mathcal{A}(\mathcal{D}')|\\
\text{s.t. } ||\mathcal{D} - \mathcal{D}'||_1 = 1,
\end{aligned}$$

where the $L1$ norm of a data set indicates how many individuals have been added or removed from a data set. To compute the local sensitivity $S_{\text{mean(z)}}$ of the mean of a vector $z = \begin{pmatrix} z_1 & \dots & z_n\end{pmatrix}' \in \mathbb{R}^n$, we denote the maximum absolute deviation of any component of z from the mean by z^*

$$z^* = \arg\max_{z_j\in\{z_1,\dots,z_n\}}\left|z_j - \frac{1}{n}\sum_{i=1}^{n} z_i\right|.$$

The data set $\mathcal{D}$ equals the vector z and removing one entry from z or adding an entry to z yields the neighboring data set $\mathcal{D}'$. The local sensitivity of the mean becomes therefore

$$\begin{aligned}
S_{\text{mean(z)}} &= \max_{z_j\in\{z_1,\dots,z_n\}}\left|\frac{1}{n}\sum_{i=1}^{n} z_i - \frac{1}{n-1}\sum_{\substack{i=1\\ i\neq j}}^{n} z_i\right|\\
&= \max_{z_j\in\{z_1,\dots,z_n\}}\left|\frac{n-1}{n(n-1)}\sum_{i=1}^{n} z_i - \frac{n}{n(n-1)}\sum_{\substack{i=1\\ i\neq j}}^{n} z_i\right|\\
&= \max_{z_j\in\{z_1,\dots,z_n\}}\left|\frac{n\sum_{i=1}^{n} z_i - \sum_{i=1}^{n} z_i - n\sum_{\substack{i=1\\ i\neq j}}^{n} z_i}{n(n-1)}\right|\\
&= \max_{z_j\in\{z_1,\dots,z_n\}}\frac{1}{(n-1)}\left|z_j - \frac{1}{n}\sum_{i=1}^{n} z_i\right|\\
&= \frac{1}{(n-1)}\max_{z_j\in\{z_1,\dots,z_n\}}\left|z_j - \frac{1}{n}\sum_{i=1}^{n} z_i\right|\\
&= \frac{1}{(n-1)}\left|z^* - \frac{1}{n}\sum_{i=1}^{n} z_i\right|.
\end{aligned}$$

For the local sensitivity $S_{\text{cov(w,z)}}$ of the covariances for z and another vector $w = \begin{pmatrix} w_1 & \dots & w_n\end{pmatrix}' \in \mathbb{R}^n$, the data $\mathcal{D}$ equals the matrix $\begin{pmatrix} w & z\end{pmatrix}$. The optimization problem becomes therefore

$$\begin{aligned}
S_{\text{cov(w,z)}} = \max_{(w_j,z_j)\in\{(w_i,z_i)\}_{i=1}^{n}}\Bigg|\frac{1}{n-1}\sum_{i=1}^{n}\left(z_i - \frac{1}{n}\sum_{k=1}^{n} z_k\right)w_i -\\
\frac{1}{n-2}\sum_{\substack{i=1\\ i\neq j}}^{n}\left(z_i - \frac{1}{n-1}\sum_{\substack{k=1\\ k\neq j}}^{n-1} z_k\right)w_i\Bigg|,
\end{aligned}$$

which we solve in our simulation study by evaluating all possible outcomes of the absolute value when removing any (w_j, z_j).

Supplementary Figures

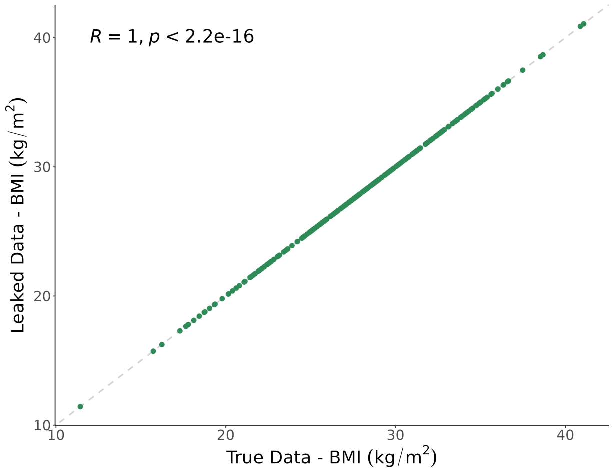

Fig. 6. Leakage results for TensorFlow Federated are shown. The true data values from the first server of the CNSIM data set are plotted against the corresponding leaked data provided by the Covariance-Based Attack Algorithm.
